# Clavicle hook plate versus distal clavicle locking plate for Neer type II distal clavicle fractures

**DOI:** 10.1186/s13018-019-1518-x

**Published:** 2019-12-30

**Authors:** Liang Li, Tian-yan Li, Peichao Jiang, Guizhen Lin, Hongxiao Wu, Xiaochuan Han, Xuezhong Yu

**Affiliations:** Department of Orthopaedics, Dongying People’s Hospital, No. 317 Dongcheng South First Road, Dongying, 257091 Shandong China

## Abstract

**Background:**

The purpose of this meta-analysis was to compare clavicle hook plates versus distal clavicle locking plates for the treatment of Neer type II distal clavicle fractures.

**Methods:**

PubMed (1996 to January 2019), Embase (1980 to January 2019), Web of Science (1990 to January 2019), the Cochrane Library (January 2019), and the China National Knowledge Infrastructure (January 2019) were systematically searched without language restrictions for literature retrieval. The Constant-Murley shoulder joint function score at 3 and 6 months after the operation and the postoperative complications after the operation (shoulder joint pain, abduction restriction, fracture delay healing, subacromial impingement) were the outcomes. Stata 12.0 was used for the meta-analysis.

**Results:**

A total of 9 clinical trials involving 446 patients were finally included in this meta-analysis. The results showed that the improvement in the Constant-Murley shoulder joint function score in the distal locking plate group was better than that in the clavicle hook plate group at 3 and 6 months after the operation (*P* < 0.05). There were fewer cases of shoulder joint pain and restricted shoulder abduction range of motion in the distal locking plate group, and the difference was statistically significant (*P* < 0.05). There were no statistically significant differences in fracture delay healing and subacromial impingement between the two groups (*P* > 0.05).

**Conclusion:**

Compared with the clavicular hook plate, the distal clavicle locking plate for the treatment of Neer type II distal clavicle fractures is associated with better shoulder function recovery and fewer complications related to pain and abduction restriction.

## Introduction

Clavicular fractures are common and typically occur in young patients, leading to a burden for this active population [1, 2]. Clavicle fractures are categorized as proximal, midshaft, or distal fractures. Although distal clavicle fractures represent only 15–28% of clavicle fracture cases, they constitute 30–45% of cases of nonunion [3–5]. Therefore, surgical management is recommended for all unstable distal clavicle fractures [6]. Controversy exists regarding the optimal treatment for vertically unstable Neer type II lateral clavicle fractures [7].

Strategies for the treatment of clavicular fractures include coracoclavicular fixation (sutures such as a tight rope or endobutton and screw) and fracture fixation devices (clavicular hook plate, clavicular locking plate and screw with lateral extension, tension band wiring and transacromial pinning with Kirschner wires fixation) [8, 9]. Clavicular hook plates and clavicular locking plates are two common internal fixation methods for treating clavicular fractures. However, the efficacy and safety of clavicular hook plates and clavicular locking plates in the treatment of clavicular fractures remains controversial. Tan et al. [10] found that the clavicular locking plate was associated with an increase in Constant-Murley scores at 3 months compared with the clavicular hook plate, which indicated that the clavicular locking plate was a better choice of treatment for clavicular fractures. However, Xiong et al. [11] revealed that there was no statistically significant difference between the clavicular hook plate and the clavicular locking plate in terms of the Constant-Murley scores.

However, there have been no systematic, quantitative evaluations comparing the two methods. In this systematic review and meta-analysis, we included relevant studies to compare the clinical outcomes of clavicular hook plates and clavicular locking plates in individuals with clavicular fractures to provide some evidence for clinical decision making.

We performed a meta-analysis that compared the clinical efficacy and safety of clavicular hook plates and clavicular locking plates for the treatment of Neer type II distal clavicle fractures.

## Materials and methods

This meta-analysis was conducted based on the recommendations in the Cochrane Handbook for Systematic Reviews of Interventions and was written in accordance with the PRISMA (Preferred Reporting Items for Systematic Reviews and Meta-analyses) checklist [12]. The meta-analysis extracted relevant data from published studies, so an ethics review approval was not required.

### Search strategy

**Fig. 1:**
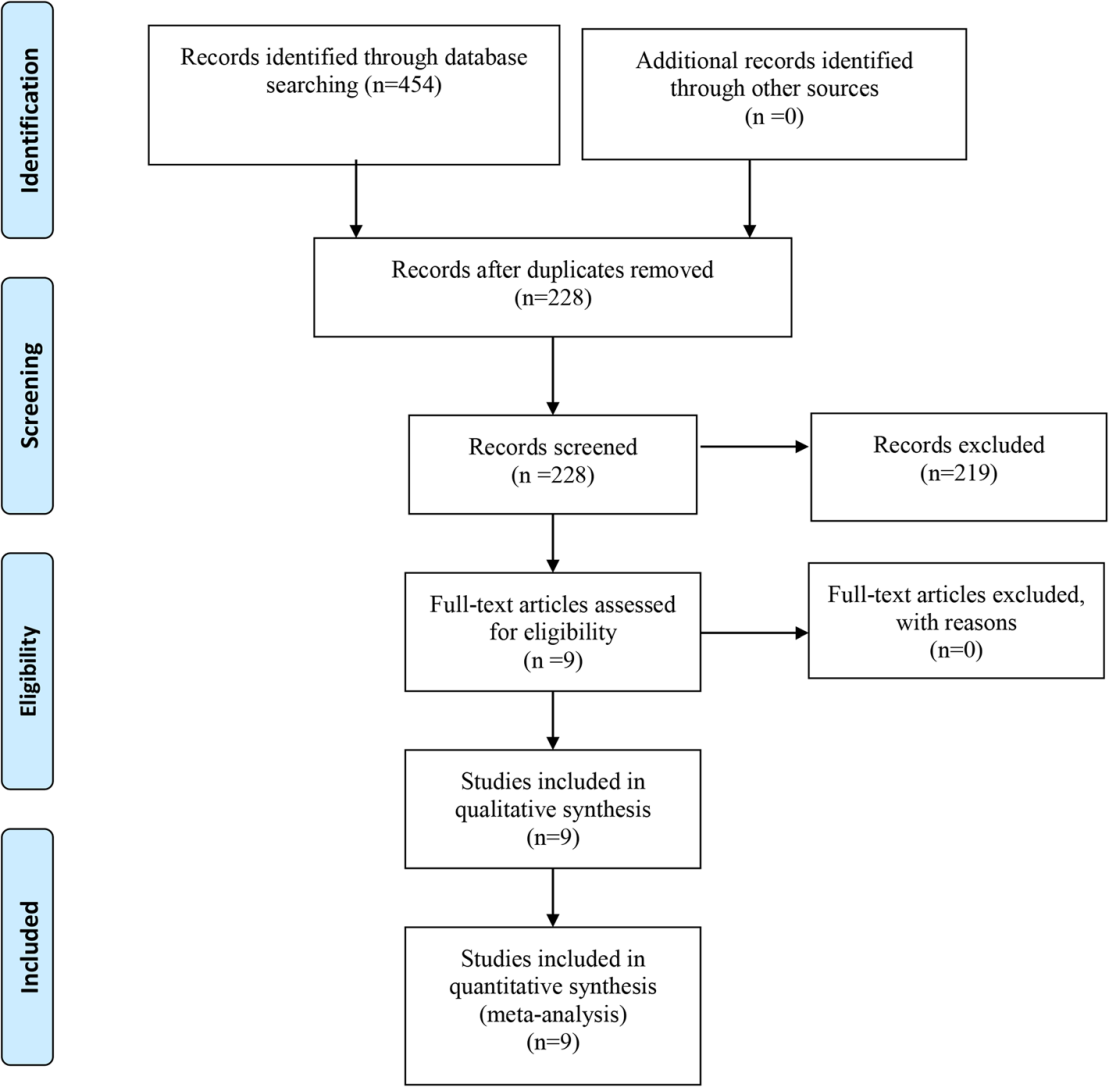
Flow diagram of the study selection process

PubMed (1996 to January 2019), Embase (1980 to January 2019), Web of Science (1990 to January 2019), the Cochrane Library (January 2019), and the China National Knowledge Infrastructure (January 2019) were systematically searched. The references of all randomized controlled trials (RCTs) were searched for additional studies related to Neer type II distal clavicle fractures. The keywords used were “distal clavicle fractures,” “clavicle fractures,” “clavicle hook plate,” and “distal clavicle locking plate” in conjunction with the Boolean operators “AND” or “OR”. The two authors independently reviewed the titles and abstracts of the articles to exclude significantly unrelated studies. The search procedure is presented in Fig. 1.

### Inclusion criteria and exclusion criteria

Studies were eligible for the meta-analysis if they met the following criteria: (1) participants: patients with distal clavicle fractures; (2) intervention: patients receiving a clavicle hook plate in the study group and distal clavicle locking plate in the study group; (3) outcomes: Constant-Murley scores at 3 months and 6 months, the occurrence of shoulder pain, restricted shoulder abduction range of motion cases, delayed union and acromion impingement syndrome; and (4) study design: RCT. Studies that did not include the outcome measures of interest were excluded. Letters, abstracts, comments, editorials, and studies without the full text available were also excluded. Disputes were resolved through discussion or the third reviewer was consulted.

### Data extraction

Two authors independently extracted the available data from the included studies. The data included the first author, publication date, number of patients, location of the fracture, incision type, fracture type, number of female patients, patient’s age, follow-up duration, outcomes, and study type. The primary outcome included the Constant-Murley scores at 3 months and 6 months. The secondary outcomes included the occurrence of shoulder pain, restricted shoulder abduction range of motion cases, delayed union, and acromion impingement syndrome. All extracted data were summarized in a pre-designed Microsoft Excel worksheet. Disagreements were resolved by discussion to reach a consensus.

### Quality assessment

Two authors (Liang Li and Tian-yan Li) independently evaluated the risk of bias of the included studies according to the Cochrane Handbook, which considered the following items: “random sequence generation,” “allocation concealment,” “blinding of participants and personnel,” “blinding of outcomes assessors,” “incomplete outcome data,” “selective outcome reporting,” and “other bias” [12]. The methodological quality of each included study was scored as “low bias,” “unclear bias,” or “high bias.”

### Data analysis and statistical methods

The current meta-analysis was conducted using Stata 12.0 (Stata Corp., College Station, TX). For the continuous outcomes, the mean difference (MD) with a 95% confidence interval (CI) was used. For the discontinuous outcomes, relative risk (RR) with a 95% CI was used. The statistical heterogeneity was assessed by the *P* value and *I*^2^ statistic using the chi-squared test. If *I*^2^ > 50% or *P* < 0.05, the studies were considered to demonstrate significant heterogeneity, and a random-effects model was used; otherwise, a fixed-effects model was used. This meta-analysis also used a funnel plot of the urinary tract infection data to independently assess publication bias.

## Results

### Search results

A total of 454 studies were identified through the initial search, and 226 papers were excluded because they were duplicates. In the next stage, 216 papers were excluded after the titles and abstracts were read. Therefore, 9 RCTs [10, 11, 13–19] were included on the basis of the inclusion criteria after the full texts were read; the RCTs included 230 patients in the clavicle hook plate group and 216 patients in the distal clavicle locking plate group.

### Study characteristics

The baseline characteristics of the included studies are summarized in Table 1. The publication year ranged from 2011 to 2014. Seven studies used the beach chair position, and the remaining two studies did not state the position. All of the included studies used a longitudinal incision for the operation. The included patients had Neer type II fractures. The proportion of female patients ranged from 26.4 to 62.4%. The age of the included patients ranged from 36.8 to 52 years. All of the studies were RCTs.

**Table 1:** General characteristic of the included studies

Author	No. of patients (I/C)	Position	Incision	Fracture type	Female (%)	Age (year)	Follow-up	Outcomes	Study
Dai 2011	25 vs 20	Beach chair	Longitudinal	Neer IIB	32.5	45.6	3 months	1, 2, 3, 4, 5	RCT
Wu 2012	12 vs 13	Beach chair	Longitudinal	Neer II	44.4	36.8	8.66 months	1, 2, 3, 4, 5, 6	RCT
Xiong 2012	21 vs 13	Beach chair	Longitudinal	Neer II	50.8	41.26	24 months	1, 2, 3, 4, 6	RCT
Tan 2012	23 vs 19	Beach chair	Longitudinal	Neer II	35.7	43.7	NS	1, 2, 3, 5, 6	RCT
Zhang 2013	35 vs 35	NS	Longitudinal	Neer II	26.4	52	15.2 months	1, 2, 3, 4, 5, 6	RCT
Du 2014	24 vs 28	NS	Longitudinal	Neer II	39.8	40.5	NS	1, 2, 3, 4, 5, 6	RCT
Hu 2014	30 vs 32	Beach chair	Longitudinal	Neer II	56.8	39	9 months	1, 2, 3, 4, 5, 6	RCT
Zhu 2014	26 vs 20	Beach chair	Longitudinal	Neer II	62.4	41.13	27.5 months	1, 2, 3, 6	RCT
Dou 2014	34 vs 36	Beach chair	Longitudinal	Neer II	44.0	52	12 months	1, 2, 3, 4, 5, 6	RCT

*1* Constant-Murley scores at 3 months; *2* Constant-Murley scores at 6 months; *3*, the occurrence of shoulder pain; *4*, the occurrence of shoulder joint abduction limited cases, *5*, the occurrence of delayed union; *6*, the occurrence of acromion impingement syndrome

### Risk of bias in the included studies

**Fig. 2:**
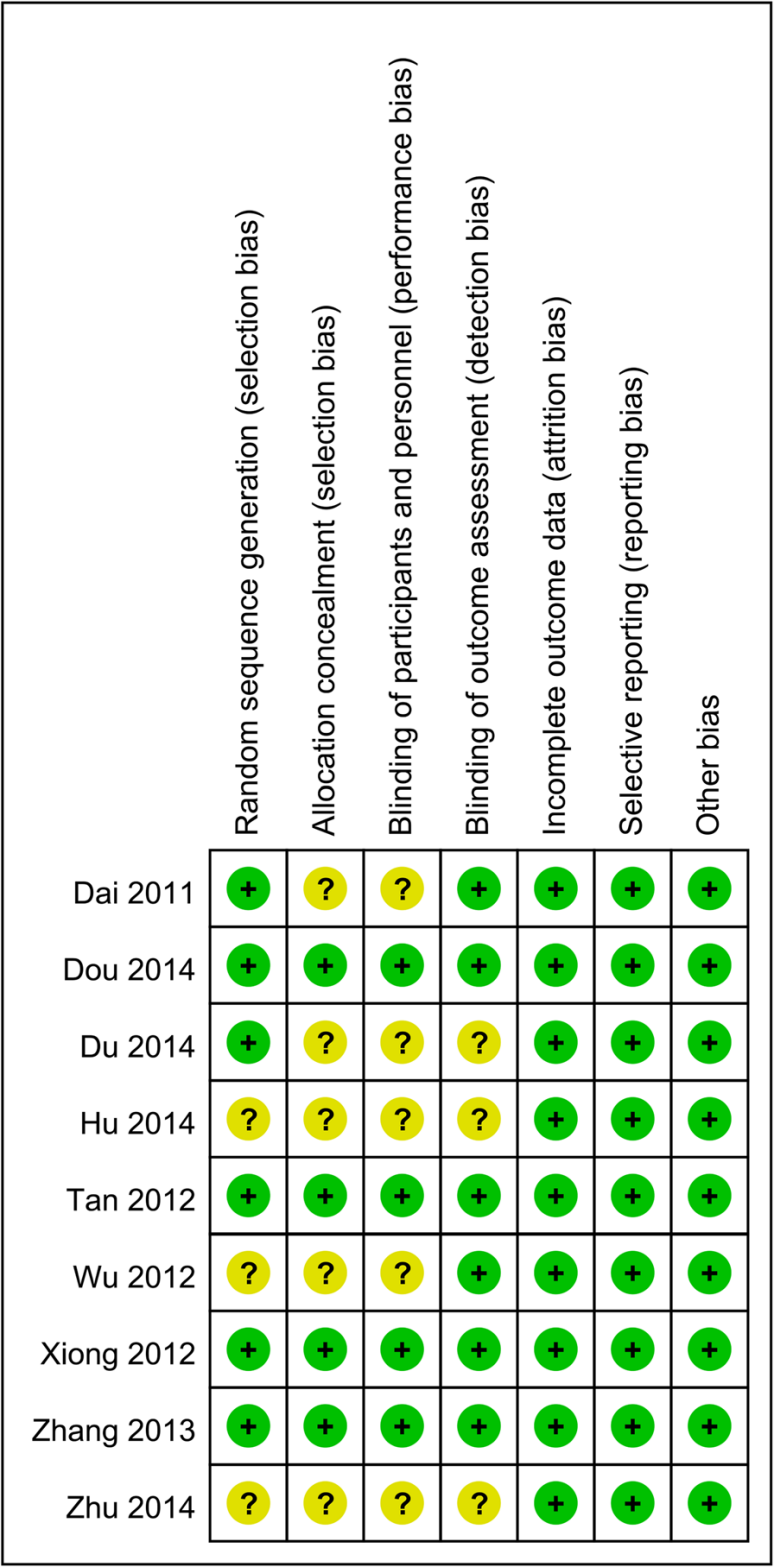
Risk of bias summary

**Fig. 3:**
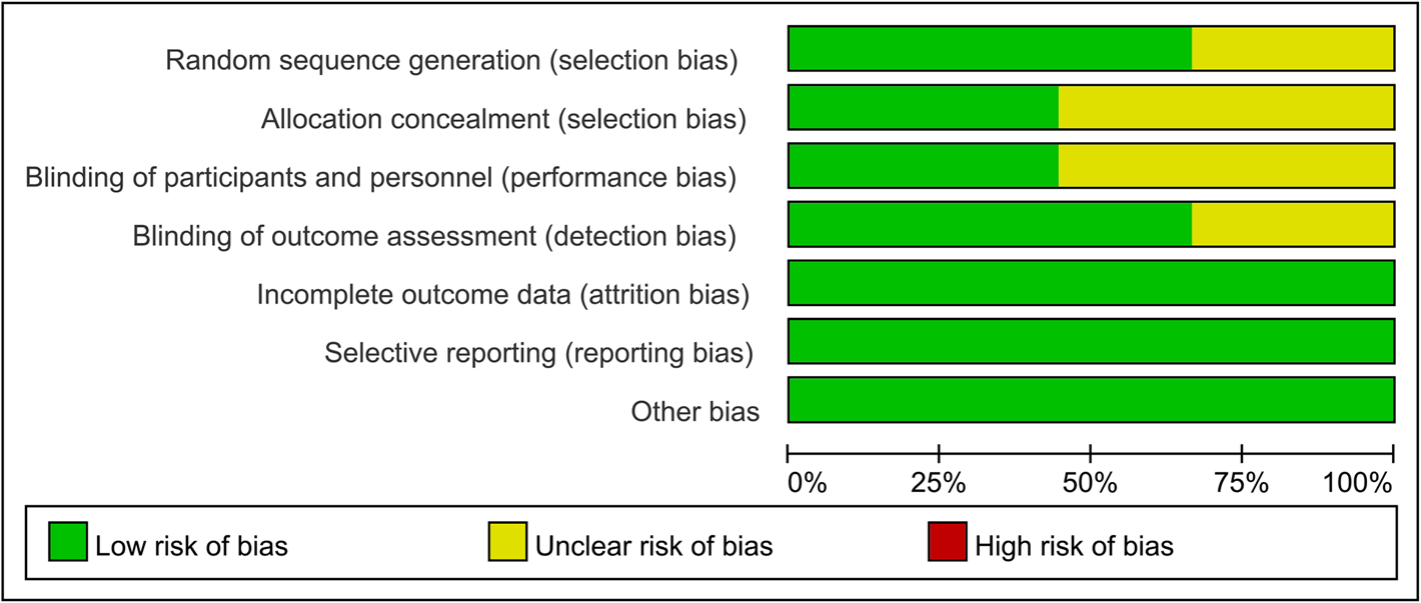
Risk of bias graph

Figures 2 and 3 show the risk of bias assessment summary and risk of bias graph, respectively. Six RCTs described using random sequence generation, and 4 studies reported using allocation concealment and blinding of the participants and personnel. Six studies reported blinding of the outcome assessors. The attrition bias, reporting bias, and other bias categories were all associated with a low risk of bias.

### Meta-analysis results

#### Constant-Murley scores at 3 months

**Fig. 4:**
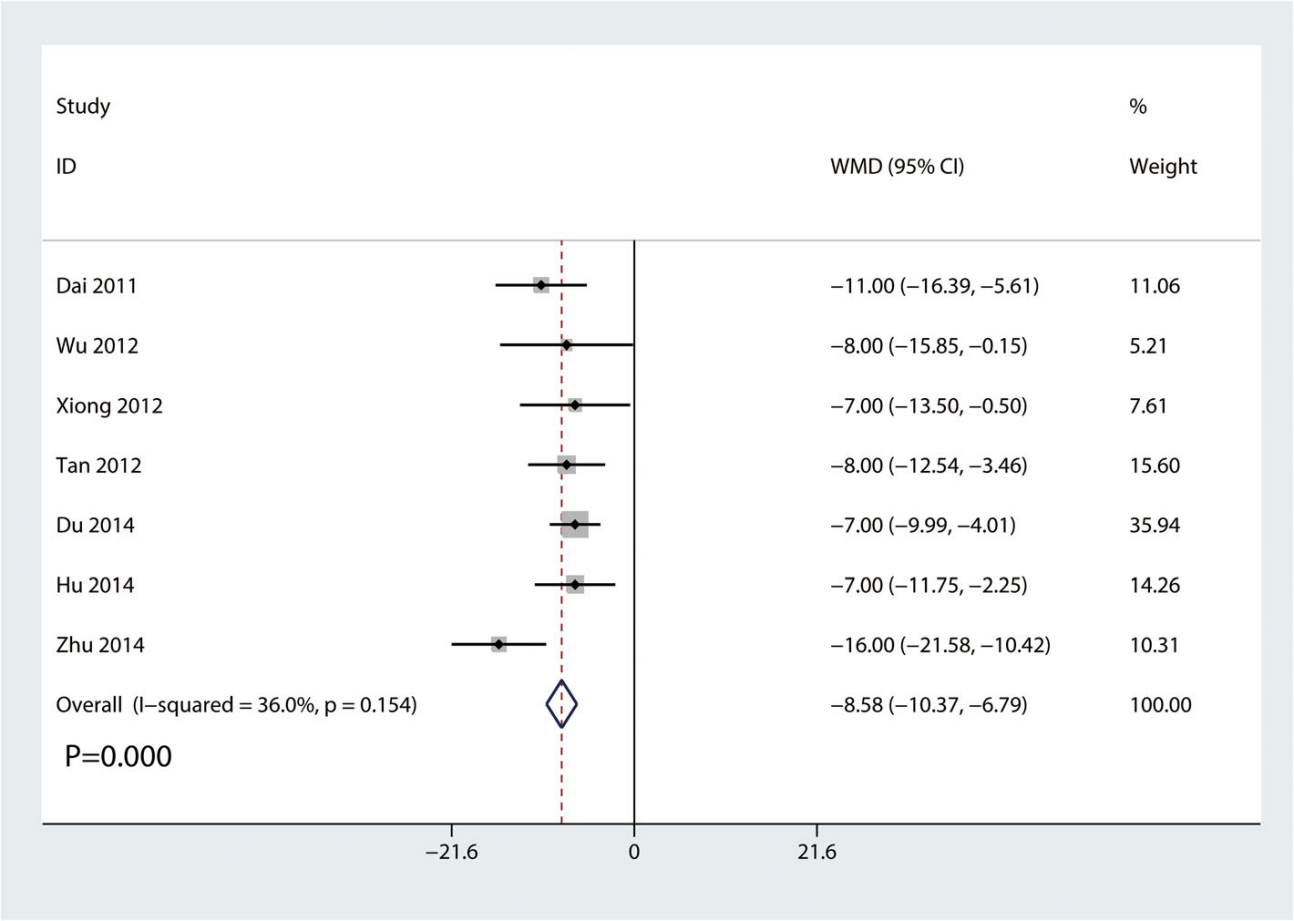
Forest plot for the comparison of Constant-Murley scores at 3 months between clavicle hook plate and distal clavicle locking plate groups

Seven trials involving 306 participants reported Constant-Murley scores at 3 months. Compared with the clavicle hook plate, the distal clavicle locking plate was associated with higher Constant-Murley scores at 3 months (MD, − 8.58; 95% CI, − 10.37 to − 6.79; *P* = 0.000; Fig. 4). Little statistical heterogeneity was observed across trials (*I*^2^ = 36.0%).

#### Constant-Murley scores at 6 months

**Fig. 5:**
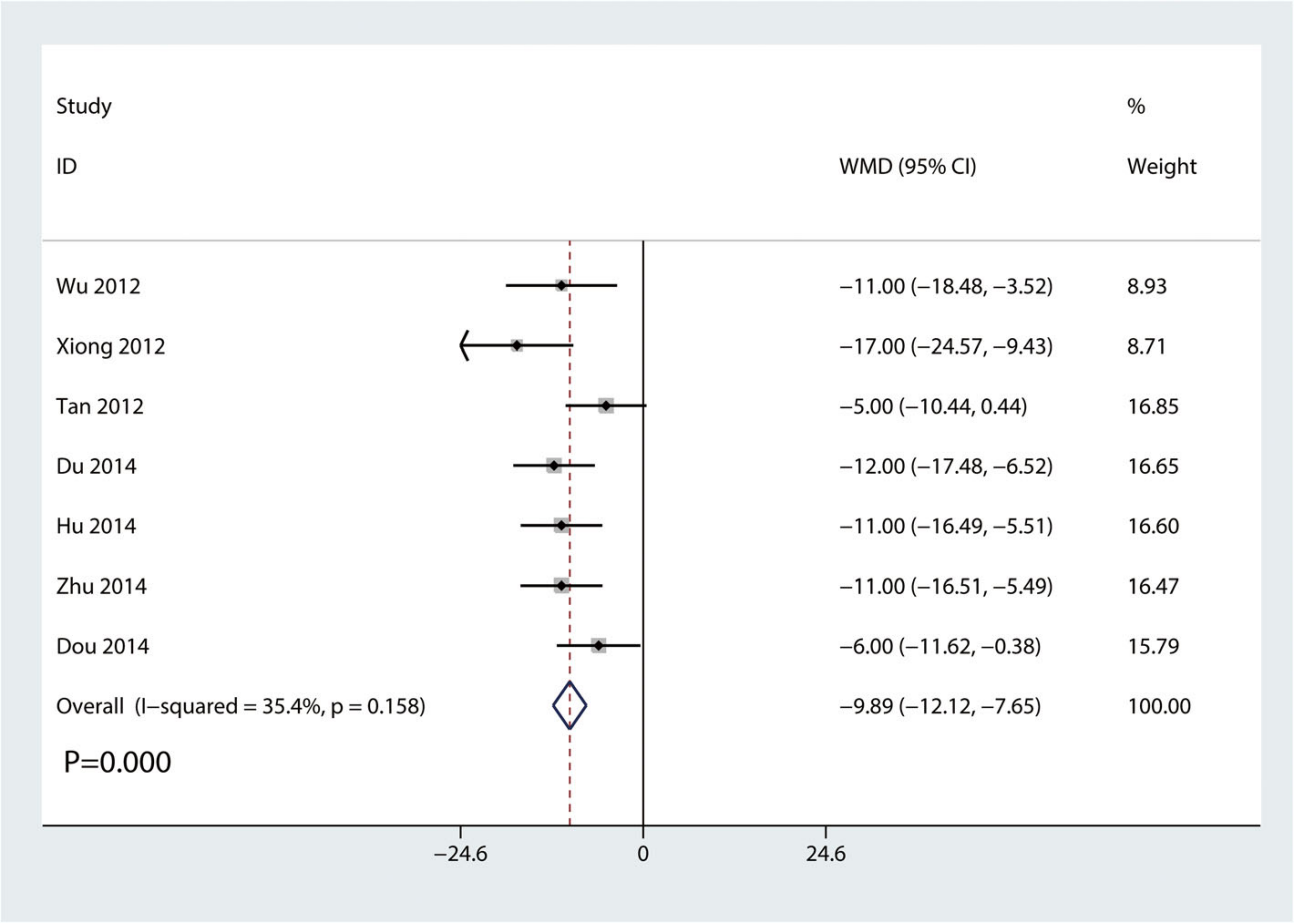
Forest plot for the comparison of Constant-Murley scores at 6 months between clavicle hook plate and distal clavicle locking plate groups

Seven trials involving 331 participants reported Constant-Murley scores at 6 months. Compared with the clavicle hook plate, the distal clavicle locking plate was associated with higher Constant-Murley scores at 6 months (MD, − 9.89; 95% CI, − 12.12 to − 7.65; *P* = 0.000; Fig. 5). Little statistical heterogeneity was observed across trials (*I*^2^ = 35.4%).

#### The occurrence of shoulder pain

**Fig. 6:**
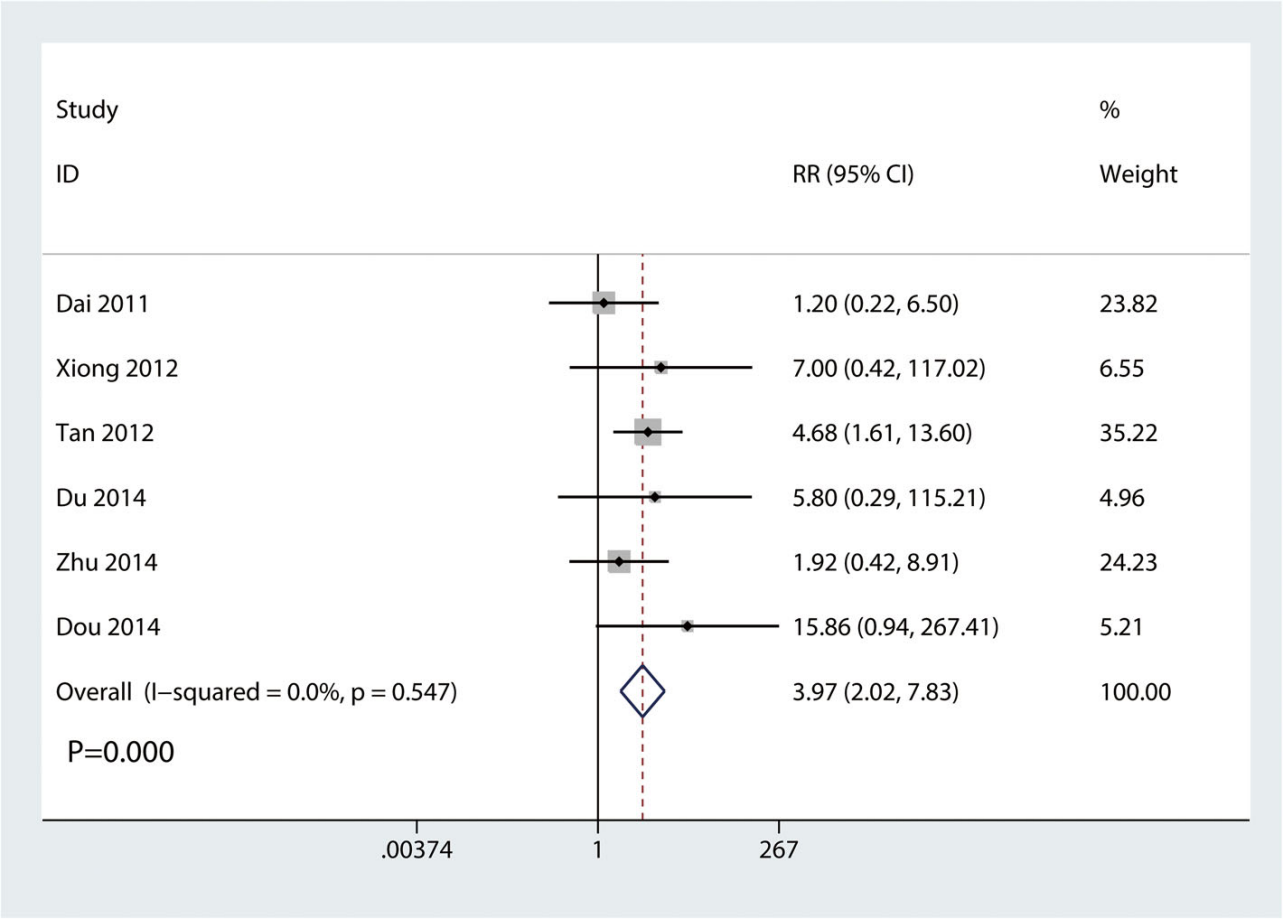
Forest plot for the comparison of the occurrence of shoulder pain between the clavicle hook plate and distal clavicle locking plate groups

Six trials involving 289 participants reported the occurrence of shoulder pain. Compared with the clavicle hook plate, the distal clavicle locking plate was associated with a decrease in the occurrence of shoulder pain (RR, 3.97; 95% CI, 2.02 to 7.83; *P* = 0.000; Fig. 6). No statistical heterogeneity was observed across trials (*I*^2^ = 0.0%).

#### Restricted shoulder abduction range of motion cases

**Fig. 7:**
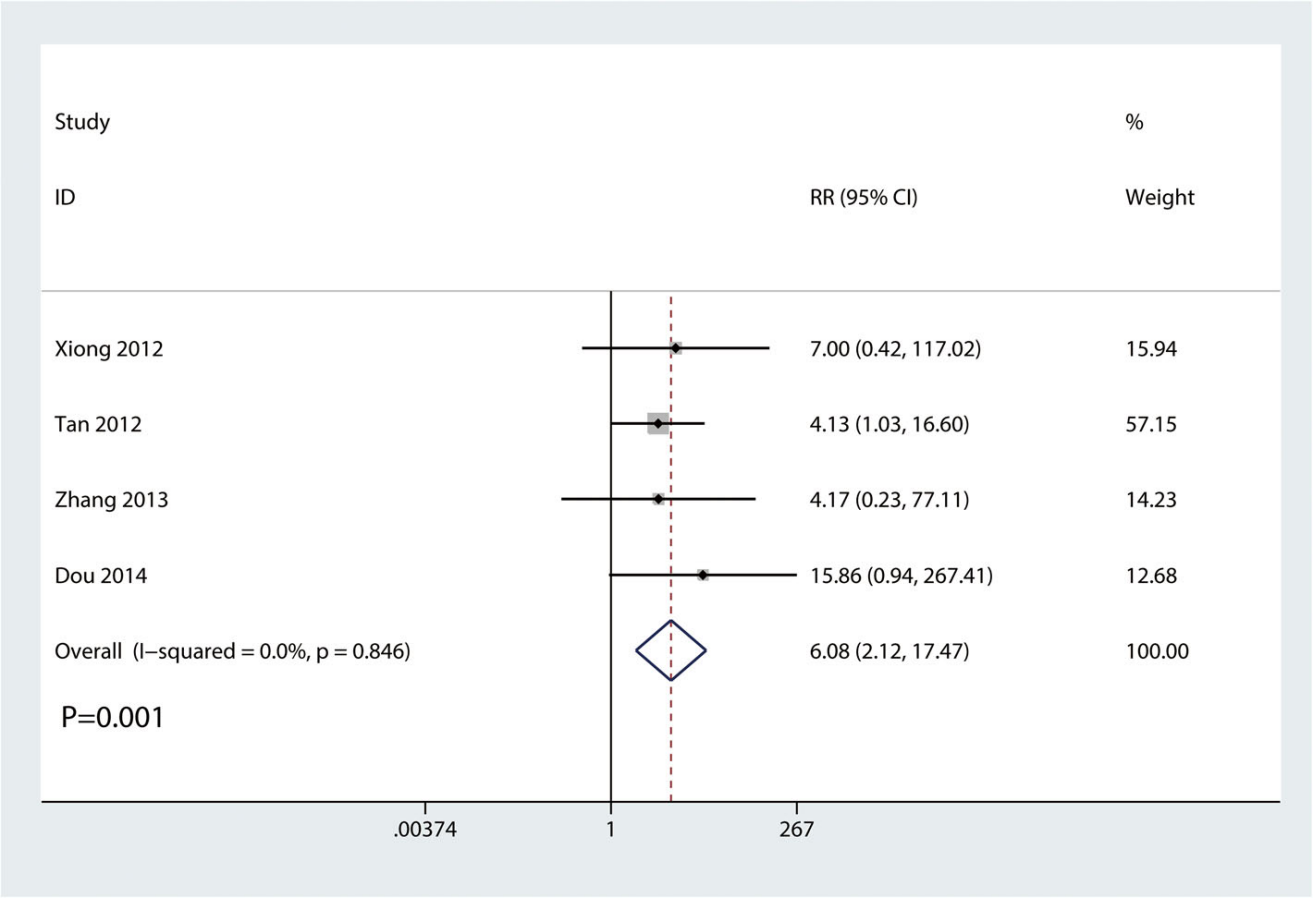
Forest plot for the comparison of the occurrence of shoulder joint abduction limited cases between clavicle hook plate and distal clavicle locking plate groups

Four trials involving 166 participants reported a restricted shoulder abduction range of motion cases. Compared with the clavicle hook plate, the distal clavicle locking plate was associated with a decrease in the number of restricted shoulder abduction range of motion cases (RR, 6.08; 95% CI, 2.12 to 17.47; *P* = 0.001; Fig. 7). No statistical heterogeneity was observed across trials (*I*^2^ = 0.0%).

#### The occurrence of delayed union

**Fig. 8:**
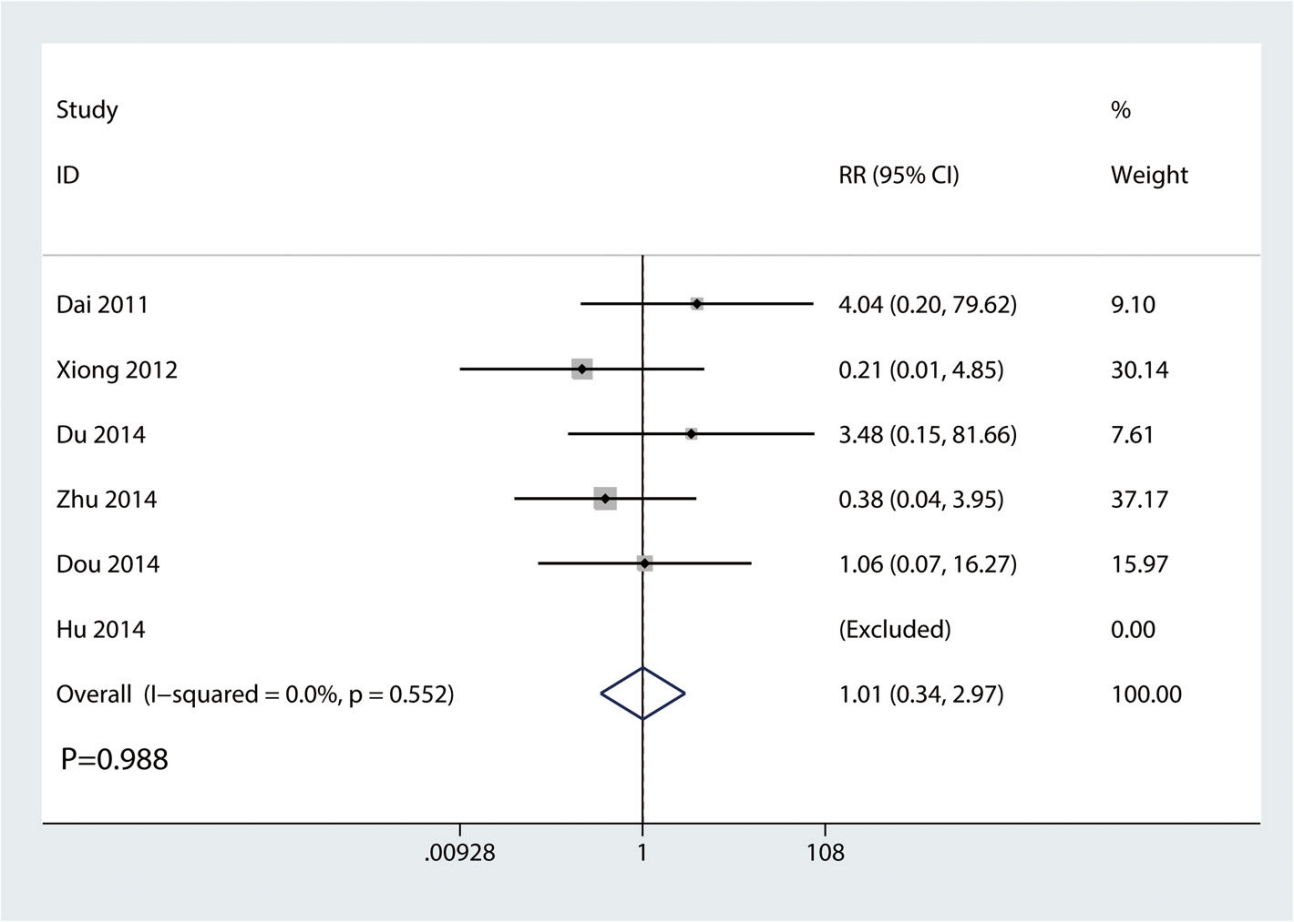
Forest plot for the comparison of the occurrence of delayed union between clavicle hook plate and distal clavicle locking plate groups

Six trials involving 292 participants reported the occurrence of delayed union. There was no statistically significant difference between the clavicle hook plate and distal clavicle locking plate groups in terms of the occurrence of delayed union (RR, 1.01; 95% CI, 0.34 to 2.97; *P* = 0.988; Fig. 8). No statistical heterogeneity was observed across trials (*I*^2^ = 0.0%).

#### The occurrence of acromion impingement syndrome

**Fig. 9:**
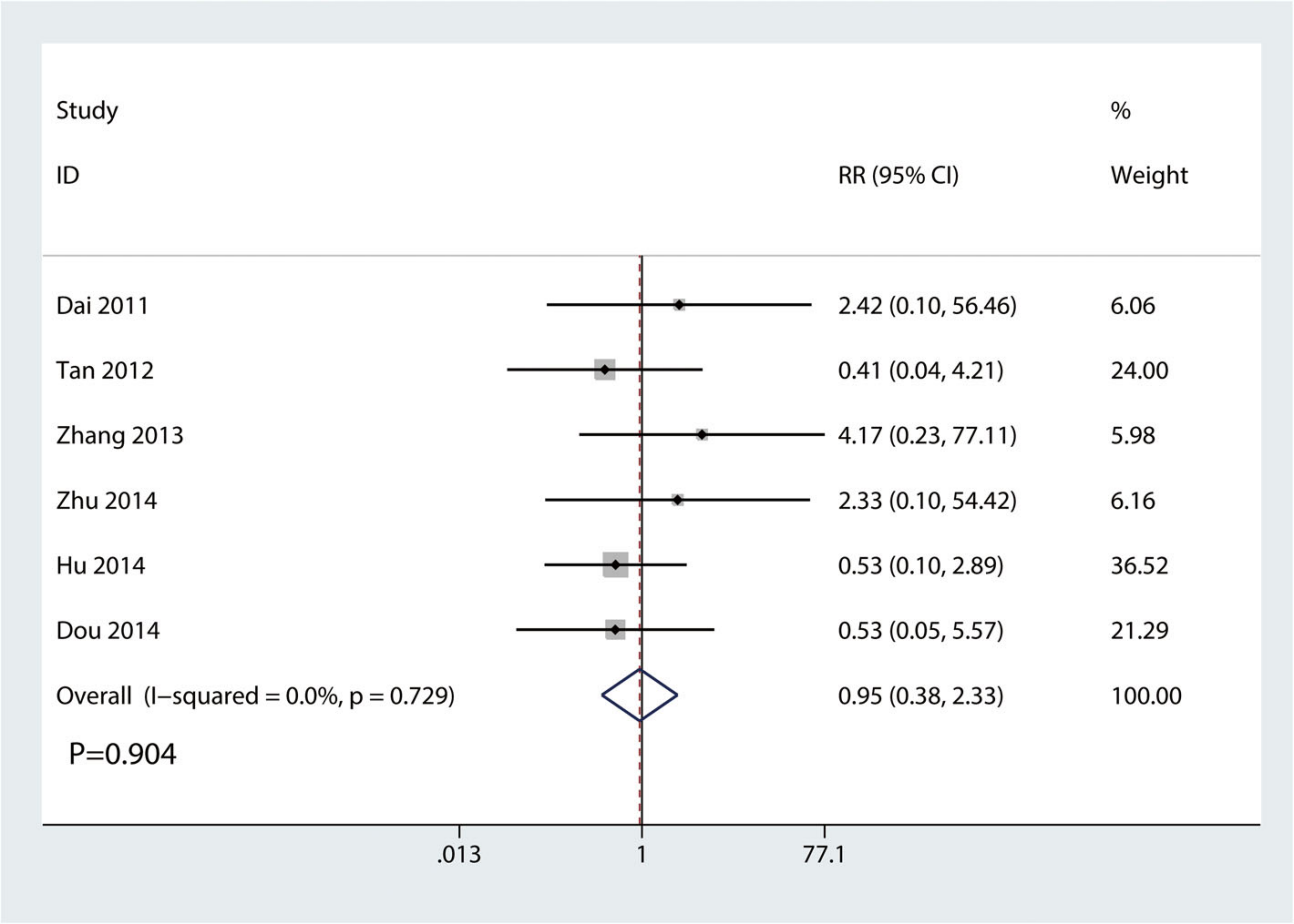
Forest plot for the comparison of the occurrence of acromion impingement syndrome between clavicle hook plate and distal clavicle locking plate groups

Six trials involving 168 participants reported the occurrence of acromion impingement syndrome. There was no statistically significant difference between the clavicle hook plate and distal clavicle locking plate groups in terms of the occurrence of acromion impingement syndrome (RR, 0.95; 95% CI, 0.38 to 2.33; *P* = 0.904; Fig. 9). No statistical heterogeneity was observed across trials (*I*^2^ = 0.0%).

#### Sensitivity analysis and publication bias

**Fig. 10:**
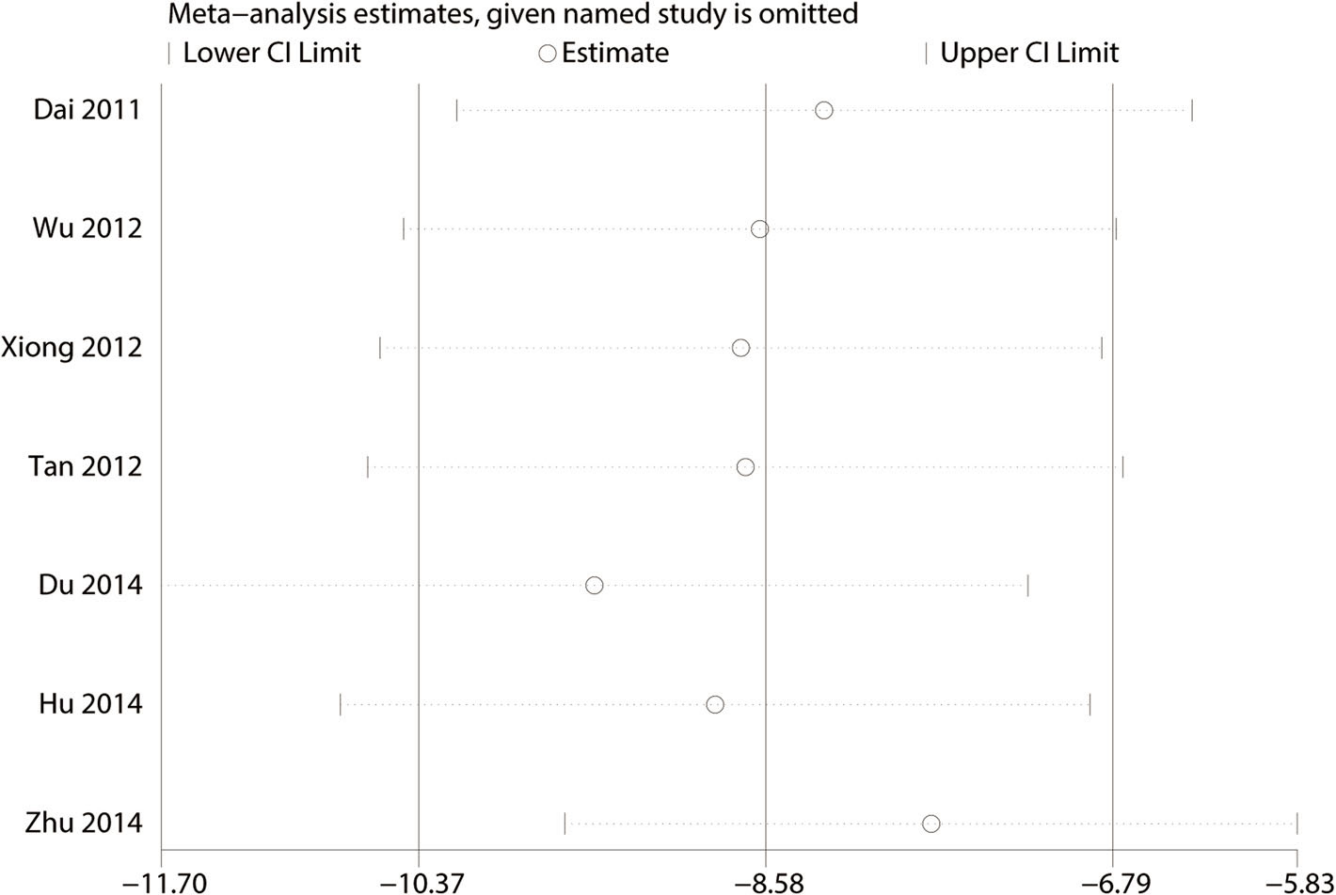
Sensitivity analysis for Constant-Murley scores at 3 months between clavicle hook plate and distal clavicle locking plate groups

A sensitivity analysis was performed by omitting included studies sequentially, and the results suggested that after removing each study sequentially, the overall effect size was not changed (Fig. 10).

**Fig. 11:**
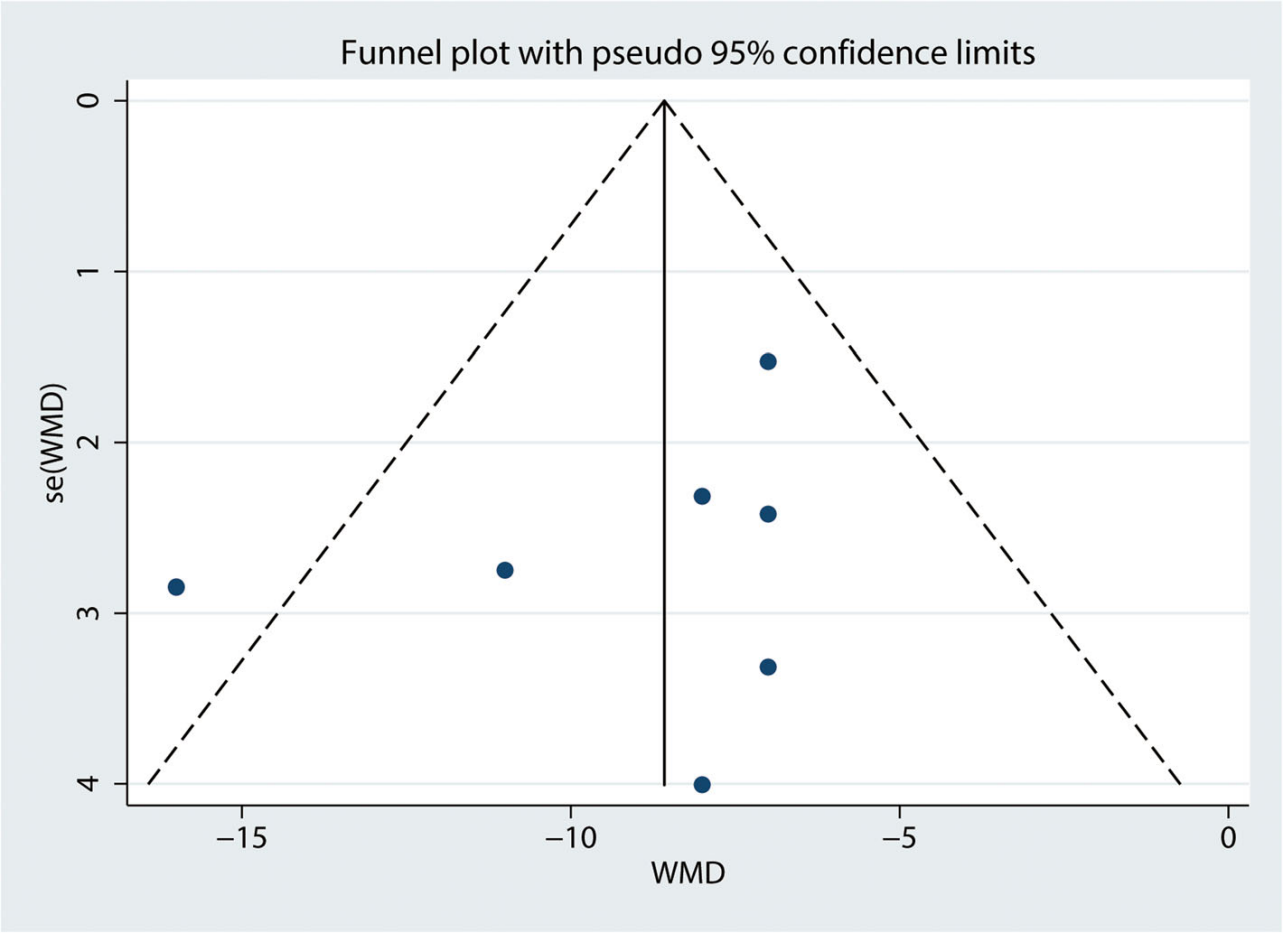
Funnel plot of the Constant-Murley scores at 3 months between clavicle hook plate and distal clavicle locking plate groups

**Fig. 12:**
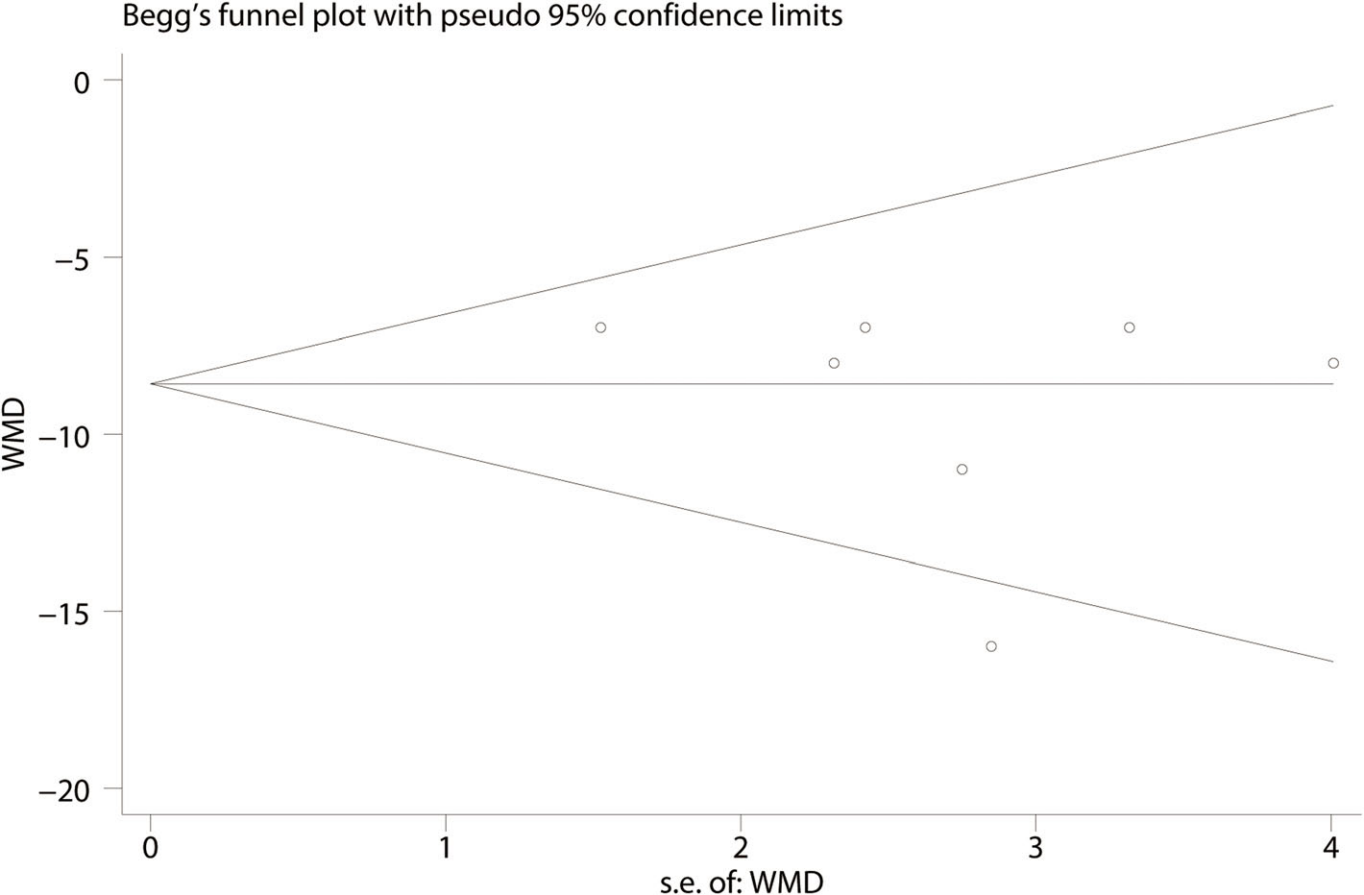
Egger’s test for Constant-Murley scores at 3 months between clavicle hook plate and distal clavicle locking plate groups

**Fig. 13:**
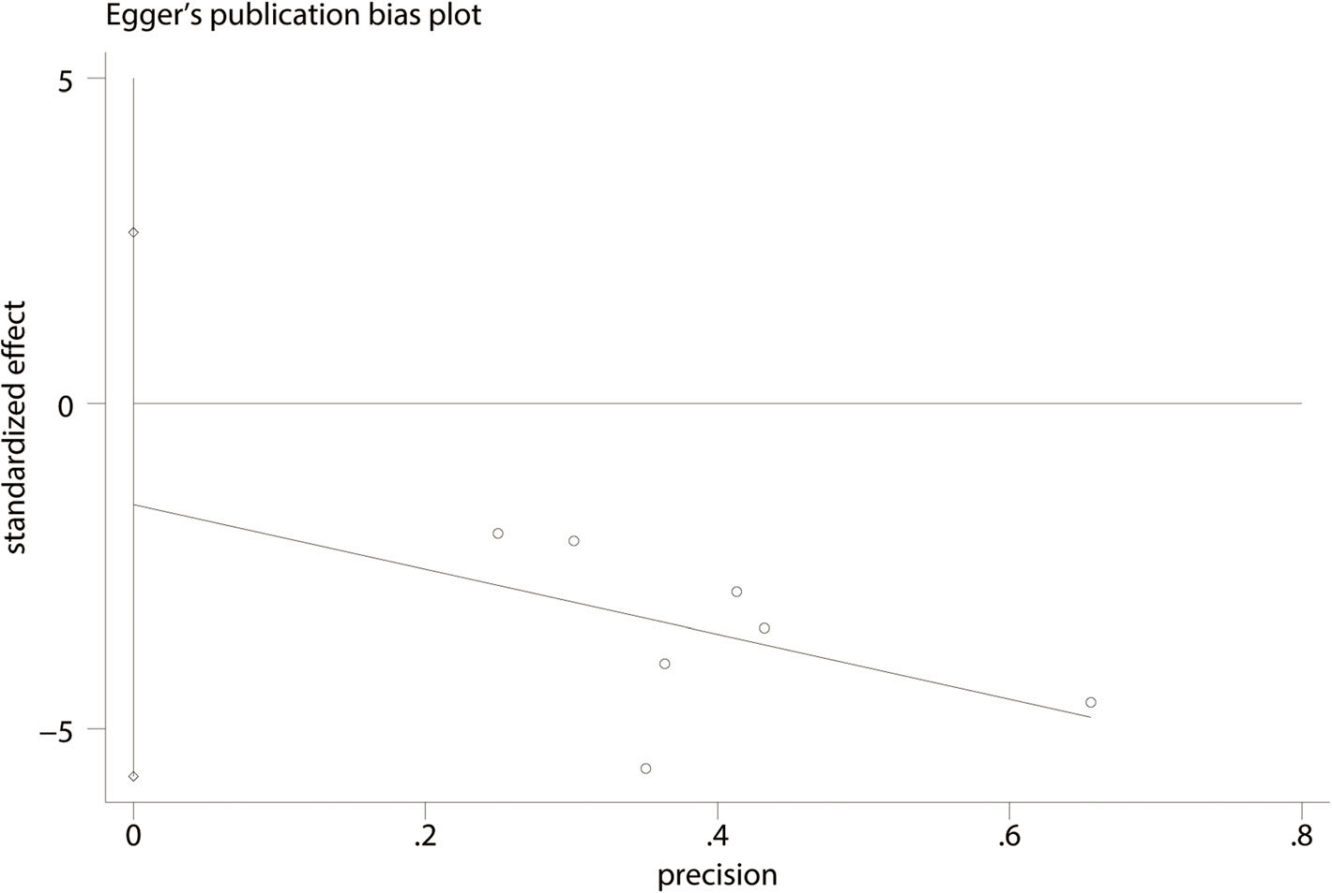
Begg’s test for Constant-Murley scores at 3 months between clavicle hook plate and distal clavicle locking plate groups

For the meta-analysis of the Constant-Murley scores at 3 months, there was no evidence of publication bias according to an inspection of the funnel plot (Fig. 11) and formal statistical tests (Egger test, *P* = 0.69, Fig. 12; Begg test, *P* = 0.73, Fig. 13).

## Discussion

### Principal findings

Compared with the clavicle hook plate, the distal clavicle locking plate was associated with increased Constant-Murley scores at 3 months and 6 months. In addition, the distal clavicle locking plate was associated with a decrease in the occurrence of shoulder pain and the number of restricted shoulder abduction range of motion cases. There was no significant difference between the clavicle hook plate and distal clavicle locking plate groups in terms of the occurrence of delayed union and the occurrence of acromion impingement syndrome. We performed a sensitivity analysis and found that after removing each study sequentially, the overall effect size was not changed.

### Relation to other systematic reviews

Only one previous systematic review and network meta-analysis comparing clavicle hook plates and distal clavicle locking plates have been published [20]. The major concern about the above meta-analysis was that they only included one potential trial that compared the Constant-Murley score between clavicle hook plates and distal clavicle locking plates. The results of that meta-analysis showed that the distal clavicle locking plate was superior to the clavicle hook plate.

In this meta-analysis, we used the Constant-Murley scores at 3 months as the primary outcome. The results showed that compared with the clavicle hook plate, the distal clavicle locking plate was associated with increased Constant-Murley scores at 3 months and 6 months. Our results were compatible with the results of previous studies [10, 21]. Zhang et al. [21] conducted a retrospective study that compared clavicle hook plates and distal clavicle locking plates for the treatment of unstable distal clavicle fractures. The results showed that both the distal clavicular locking plate and the clavicular hook plate achieved good results in the treatment of unstable distal clavicle fractures. In addition, more individuals who underwent internal fixation with a distal clavicular locking plate were able to return to their previous work within 3 months after surgery and had fewer complications than those who underwent fixation with the clavicular hook plate. Erdle et al. [22] found that the overall functional outcome was similar in the clavicle hook plate and distal clavicle locking plate groups. We performed a sensitivity analysis by sequentially omitting each study, and this analysis showed that our results were statistically reliable. Biomechanical studies have shown that the clavicle locking plate is superior to the clavicle hook plate in terms of flexion and abduction of the humerus. Because locking plate fixation maintains the biomechanics of the acromioclavicular joint, it allows some degree of early mobilization and does not require reconstruction of the coracoclavicular ligaments [23].

The treatment of Neer type II distal clavicle fractures is a controversial topic. Several studies have recommended open reduction and internal fixation for Neer type II distal clavicle fractures because these fractures tend to displace and are associated with a higher risk of nonunion compared with other clavicle fracture types. We compared the occurrence of delayed union between the clavicle hook plate and distal clavicle locking plate groups. We found that there was no significant difference between the occurrence of delayed union between the clavicle hook plate and distal clavicle locking plate groups.

We then compared the occurrence of shoulder pain and the restricted shoulder abduction range of motion cases between the clavicle hook plate and distal clavicle locking plate groups. Compared with the clavicle hook plate, the distal clavicle locking plate was associated with a decrease in the occurrence of shoulder pain and a restricted shoulder abduction range of motion cases.

### Limitation of this meta-analysis

Some limitations of this study should be noted. First, the small sample size might have affected the significant difference between the two surgical procedures. Second, most of the included studies originated from China, and more RCTs published in other countries should be conducted to confirm this conclusion. Third, our study ignored the diversity of diagnostic criteria and etiology of the disease, and further research is needed to determine whether these conclusions apply to patients with the various other types of distal clavicle fractures.

## Conclusion

Compared with the clavicular hook plate, the distal clavicle locking plate for the treatment of Neer type II distal clavicle fractures is associated with better shoulder function recovery and fewer complications related to pain and abduction restriction. In view of the heterogeneity and different follow-up times, whether these conclusions are applicable should be further assessed in future studies.

## Data Availability

We state that the data will not be shared since all the raw data are present in the figures included in the article.

## Abbreviations

CIs: Confidence interval
MD: Mean difference
PRISMA: Preferred reporting items for systematic reviews and meta-analyses
RCTs: Randomized controlled trials
RR: Risk ratio
